# The Influence of Functional Endoscopic Sinus Surgery on Sleep Related Outcomes in Patients with Chronic Rhinosinusitis

**DOI:** 10.1155/2019/7951045

**Published:** 2019-06-02

**Authors:** Rong-San Jiang, Kai-Li Liang

**Affiliations:** ^1^Department of Medical Research, Taichung Veterans General Hospital, Taiwan; ^2^Department of Otolaryngology, Taichung Veterans General Hospital, Taiwan; ^3^School of Medicine, Chung Shan Medical University, Taiwan; ^4^Rong Hsing Research Center For Translational Medicine, National Chung Hsing University, Taichung, Taiwan; ^5^Faculty of Medicine, National Yang-Ming Medical University, Taipei, Taiwan

## Abstract

**Purpose:**

Chronic rhinosinusitis (CRS) patients often complain of nasal obstruction, which may cause sleep impairment for them. The goal of this study was to investigate the influence of functional endoscopic sinus surgery (FESS) on sleep related outcomes in CRS patients.

**Materials and Methods:**

CRS patients who received FESS were included in this study. Prior to FESS and 3 months after surgery the patients were asked about the severity of nasal obstruction and completed the 20-item Sinonasal Outcome Test (SNOT-20), along with the Epworth Sleepiness Scale (ESS) questionnaire. Endoscopic examination, acoustic rhinometry, and polysomnography were performed in all patients. They were divided into four groups according to their preoperative apnea hypopnea index (AHI) scores: nonobstructive sleep apnea syndrome (non-OSAS), mild OSAS, moderate OSAS, and severe OSAS.

**Results:**

A total of 96 subjects completed the study. The scores of the sleep domain of the SNOT-20 and ESS decreased in all of the AHI groups, with the exception of the severe OSAS group, after FESS. A reduction in the AHI of less than 5 was achieved in 9 patients (13.2%) after FESS.

**Conclusions:**

Our results showed that FESS improved sleep quality in CRS patients, except those with severe OSAS, and a preoperative lower AHI was the only significant predictor of post-FESS OSAS outcome.

## 1. Introduction

Sleep impairment is a common symptom in patients with chronic rhinosinusitis (CRS) [1–3]. Alt et al. reported a 75% prevalence of poor sleep quality in 268 CRS patients, as measured by the Pittsburg Sleep Quality Index instrument [4]. The etiology of sleep dysfunction in CRS is multifactorial. Although CRS patients usually experience nasal obstruction, it has been suggested that CRS is associated with the release of proinflammatory cytokines, which may also result in sleep impairment [5].

Nasal surgery, including septomeatoplasty, turbinate surgery, or functional endoscopic sinus surgery (FESS), which aims to reduce upper airway resistance, has been reported to benefit sleep quality [6–9]. Sukato et al. [8] conducted a meta-analysis regarding the effect of FESS on obstructive sleep apnea syndrome (OSAS). They discovered only 7 studies showing that FESS could benefit sleep quality and possibly improve apnea hypopnea index (AHI), although the results displayed high heterogeneity among studies. More research is needed to establish whether or not FESS could improve sleep problems in CRS patients. The aims of this study were to research the influence of FESS on sleep problems in CRS patients and to identify predictive factors of AHI outcomes in CRS patients with OSAS after FESS.

## 2. Materials and Methods

This study was approved by the Ethics Committee of Taichung Veterans General Hospital. Written consent was obtained from each patient.

### 2.1. Study Population

CRS patients who underwent bilateral primary FESS were collected between July 2010 and January 2017. Among them, those willing to undergo a one-night polysomnography (PSG) study before and 3 months after FESS were enrolled in this study. The diagnosis of CRS was based on the definition provided in the European Position Paper on Rhinosinusitis and Nasal Polyps 2012 [10]. All study subjects had a history of rhinosinusitis for a period greater than 12 weeks, and also displayed endoscopic and radiological evidence of nasal inflammation. Patients who were under the age of 20 or had a history of immunodeficiency were excluded. The surgical extension was based upon preoperative computed tomography (CT) and any mucosal inflammatory change which was found during surgery. If deviated nasal septum and/or hypertrophic turbinates impeded the nasal patency, septoplasty and/or turbinate surgery were performed concurrently with FESS. Patients who were diagnosed with OSAS according to their PSG results did not receive continuous positive airway pressure treatment during the study period.

The primary outcome of this study was the subjective sleep quality. The secondary outcome was the cure rate of OSAS by FESS. The cure of OSAS after FESS was defined as normalization of AHI, i.e., AHI less than 5 [11]. In addition, we tried to identify predictive factors of AHI outcomes in CRS patients with OSAS after FESS.

### 2.2. Assessment of Rhinosinusitis Severity

The severity of rhinosinusitis was assessed using the Taiwanese version of the Sinonasal Outcome Test-20 (SNOT-20) questionnaire, the grading of nasal obstruction, an endoscopic examination, and an acoustic rhinometry study to measure the second minimal cross-sectional area (MCA_2_) both prior to and 3 months after FESS, along with an preoperative CT.

The Taiwanese version of the SNOT-20 is a validated 20-item instrument to assess the rhinosinusitis-specific quality of life [12]. The patient grades each question from 0 to 5 (0 indicating “no problem” and 5 indicating “problem as bad as can be”). The total score ranges from 0 to 100. The severity of nasal obstruction was graded by the patient from 0 to 5, as in the SNOT-20. The endoscopic appearances were quantified on a 0 to 2-point scale according to the staging system devised by Lund and Kennedy [13]. The total score was determined by the sum of all the scores of the bilateral endoscopic findings (range 0-20). Nasal polyps were diagnosed by preoperative endoscopic examination. The preoperative CT scans of the study subjects were graded according to the Lund-Mackay staging system. The total score ranged from 0 to 24 [13].

### 2.3. Assessment of Sleep Quality

Before and 3 months after the FESS procedure, patients were assessed with a Chinese version of the Epworth Sleepiness Scale (ESS) questionnaire and underwent a one-night PSG assessment. The sleep domain of the SNOT-20 (items 12-17: difficulty falling asleep, waking up at night, lack of a good night's sleep, waking up tired, fatigue, reduced productivity, and reduced concentration) was calculated separately for the purpose of evaluating the sleep quality in CRS patients.

The ESS is an 8-item questionnaire that is a useful tool for evaluating daytime sleepiness in adults [14]. Each of the 8 items is scored from 0 to 3 with a total score ranging from 0 to 24. A score of 10 or more is considered to indicate that the patient suffers from daytime sleepiness [15]. The PSG measures important sleep variables, including AHI, snoring index, and lowest arterial oxygen saturation (SaO_2_). The AHI is defined as the sum of apneas and hypopneas per hour of sleep. Apnea is defined as a 90% decrease in airflow for 10 seconds, relative to the baseline value. Hypopnea is defined as a 50% decrease in the airflow amplitude for 10 seconds, relative to the baseline value, with a presence of arousal or oxygen desaturation of 4% [16]. Patients with an AHI of ≥ 5 but < 15 are considered to have mild OSAS, those with an AHI of ≥ 15 but < 30 to have moderate OSAS and those with an AHI ≥ 30 to have severe OSAS [16].

### 2.4. Statistical Analysis

All data are presented as mean ± standard deviation. The differences in gender and polyp status were compared among groups using Chi-Square test. The ages of patients, SNOT-20 scores, nasal obstruction scores, mean MCA_2_ of bilateral nasal cavities, endoscopic scores, CT scores, ESS scores, sleep domain scores of the SNOT-20, snoring index scores, and lowest SaO_2_ were all compared among the 4 AHI groups using the Kruskal-Wallis test. The severity of rhinosinusitis and sleep quality within each AHI group both before and after FESS were compared by the Wilcoxon signed rank test. Logistic regression was used to analyze the predictors for successful OSAS outcome after FESS. All computations were performed using SPSS version 17.0 (SPSS, Inc., Chicago, IL, USA). Two-tailed p-values < 0.05 were considered statistically significant.

## 3. Results

### 3.1. Demographic Data

Ninety-six patients (62 males and 34 females) completed the study. Their ages ranged from 21 to 84 years with a mean of 44.1 years. According to the PSG, 32 belonged to the non-OSAS group, 26 to the mild OSAS group, 26 to the moderate OSAS group, and 12 to the severe OSAS group. When demographic data were compared among the 4 AHI groups, CRS patients with moderate and severe OSAS were significantly older then the non-OSAS patients (p < 0.001 and = 0.002, respectively).

### 3.2. Preoperative Rhinosinusitis Severity and Sleep Quality

The characteristics of preoperative rhinosinusitis severity and sleep quality of the study subjects are shown in Table 1. The preoperative SNOT-20 and nasal obstruction scores were lower in the moderate and severe OSAS groups when compared with those in the non-OSAS group (p = 0.041 and 0.032 for moderate OSAS vs. non-OSAS respectively; p = 0.005 and 0.04 for severe OSAS vs. non-OSAS respectively), but the preoperative objective parameters of rhinosinusitis severity including mean MCA_2_, endoscopic score, and CT score were not significantly different among the 4 AHI groups. Although there were higher snoring index scores and lower SaO_2_ in the moderate and severe OSAS groups as compared to the non-OSAS and mild OSAS groups, the preoperative scores of ESS and sleep domain of the SNOT-20 were not significantly different among the four AHI groups.

### 3.3. Comparison of Rhinosinusitis Severity and Sleep Quality before and after FESS

The rhinosinusitis severity and sleep quality before and after FESS were compared for each group (Table 2). With the exception of patients with severe OSAS, other groups displayed significant improvement in the SNOT-20, nasal obstruction scores, MCA_2,_ and ESS after FESS. The sleep domain scores of the SNOT-20 significantly improved in both the non- and mild OSAS groups after FESS as well.

### 3.4. Predictors of Successful Outcome of OSAS after FESS

There were 9 patients with an AHI level less than 5. The preoperative characteristics of patients who had AHI levels less than 5 or not after FESS are listed in Table 3. Patients whose AHI levels were less than 5 after FESS had significantly a higher preoperative nasal obstruction score, lower preoperative snoring index scores, and AHI compared to those whose AHI levels were not less than 5 after FESS. Logistic regression was used to further analyze the predictive factors for successful outcome of OSAS after FESS (Table 4). We found that preoperative lower AHI was the only significant predictor for good sleep outcomes in CRS patients with OSAS after FESS.

### 3.5. Relationship between Changes of Sleep Outcomes, Body Weight Index (BMI), and Rhinological Parameters before and after Surgery

Liner analyses were performed to examine the relationship between changes of sleep outcomes (ESS, snoring index, AHI, and lowest SpO2), BMI, and rhinological parameters (Table 5). We found that change of ESS was significantly associated with that of SNOT-20 (P = 0.001).

## 4. Discussion

In this study, CRS patients with severe OSAS tended to have lower SNOT-20 and nasal obstruction scores than other patients with less severe OSAS, but the objective parameters of rhinosinusitis severity including mean MCA_2_, endoscopic score, and CT score were similar among different severity groups of OSAS. This might indicate that nasal resistance plays a limited role in the pathophysiology of OSAS in CRS patients due to multilevel upper airway obstruction.

After FESS, both subjective and objective parameters of rhinosinusitis severity improved in most patients, with the exception of CRS patients with severe OSAS. The scores of ESS and sleep domain of the SNOT-20 also significantly decreased, except in CRS patients with severe OSAS. We also found that the change of ESS significantly correlated with that of SNOT-22. It seemed that the sleep quality of CRS patients was improved following FESS because of decreased rhinosinusitis severity, unless they had severe OSAS. In a study by Rotenberg and Pan on patients without polyps, and in a study by Varendh et al. on patients with polyps, sleep quality also improved after FESS [7, 17]. A recent systematic review reported that FESS has demonstrated encouraging results in improving sleep function in OSAS patients [8]. The authors reported cumulative data analyses from 7 studies where FESS demonstrated a moderate to large good effect in subjective sleep quality and small improvement in objective AHI [8]. Our results are consistent with the aforementioned systemic review. The pathophysiology of OSAS is complex and includes anatomical, neuromuscular, and pulmonary factors, along with aging [18]. The mechanisms by which FESS benefits OSAS include the reduction of upper airway resistance and the avoidance of breathing through the mouth [19]. Mouth breathing usually aggravates sleep related breathing disorders [20, 21]. Ayuse et al. [22] reported that mouth breathing increased upper airway collapsibility during midazolam sedation. A study which enrolled 138 OSAS patients proved that mouth breathing resulted in reduction of oropharyngeal lumen by computed tomography scans [23]. It had been reported that oral patches for prevention open mouth breathing are useful to treat mild OSAS [24].

Some predictors of surgical success for OSAS have been reported in the literature [25]. Gislason et al. reported that preoperative lower AHI and BMI were predictors of success after uvulopalatopharyngoplasty [26]. Nevertheless, another study conducted in Sweden found that the successful rate for UPPP was solely dependent on tonsil size but not influenced by preoperative BMI, age, or gender [27]. No predictor of successful treated OSAS by FESS has been reported. Our results showed preoperative lower AHI was the only predictor of success, although we analyzed many other predictors such as nasal obstruction, polyp, and CT score.

There were some limitations in our study. First, the number of severe OSAS patients enrolled in our study was fewer than numbers of patients in the other AHI groups. Second, most of our study subjects had a BMI of less than 30, as obesity is less common in Asian countries [28]. Additionally, genetic and ethnic factors could lead to different OSAS treatment outcomes.

## 5. Conclusions

Our results showed that sleep quality in CRS patients improved following FESS. CRS patients with OSAS who had a lower preoperative AHI might concurrently acquire a successful OSAS outcome after FESS.

## Figures and Tables

**Table 1:** Comparison of preoperative characteristics when classified by apnea-hypopnea index (AHI).

Classification	Non-OSAS	Mild OSAS	Moderate OSAS	Severe OSAS	P value
	N=32	N= 26	N= 26	N=12	

Gender (Male/Female)	10/22	11/15	21/5	8/4	0.031∗ ^a^
Age (years)	36.5 ± 11.9	43.0 ± 13.1	51.5 ± 13.7	51.0 ± 12.9	<0.001∗ ^b^
SNOT-20 score	40.0 ± 17.5	37.9 ± 18.7	30.3 ± 21.2	23.1 ± 14.6	0.019∗ ^b^
Nasal obstruction score	3.4 ± 1.2	3.4 ± 1.6	2.5 ± 1.6	2.3 ± 1.7	0.029∗ ^b^
MCA_2_	0.36 ± 0.17	0.40 ± 0.15	0.42 ± 0.20	0.49 ± 0.19	0.181 ^b^
Endoscopic score	5.1 ± 1.9	5.2 ± 2.3	5.5 ± 2.4	5.1 ± 3.0	0.874 ^b^
Without polyp/with polyp	16/16	12/14	16/10	6/6	0.712 ^a^
CT score	12.0 ± 4.6	10.9 ± 5.1	12.8 ± 5.3	10.5 ± 5.2	0.588 ^b^
BMI	24.00 ± 4.49	25.30 ± 3.04	25.66 ± 5.83	29.34 ± 4.21	0.002∗ ^b^
ESS score	8.9 ± 5.2	8.4 ± 4.6	9.2 ± 4.5	5.7 ± 3.7	0.233 ^b^
Sleep-domain of SNOT-20	14.2 ± 7.8	14.4 ± 8.2	11.3 ± 8.3	10.1 ± 7.9	0.231 ^b^
Snoring index	130.05 ± 103.02	274.44 ± 102.06	322.73 ± 123.70	497.86 ± 137.30	<0.001∗ ^b^
AHI	1.89 ± 1.35	9.05 ± 2.85	19.88 ± 3.62	51.21 ± 14.93	<0.001∗ ^b^
Lowest SaO_2_ (%)	90.53 ± 3.06	86.19 ± 5.10	85.14 ± 7.73	72.68 ± 12.53	<0.001∗ ^b^

OSAS: obstructive sleep apnea syndrome; Non-OSAS: AHI < 5; mild OSAS: AHI ≥ 5 but < 15; moderate OSAS: AHI ≥ 15 but < 30; severe OSAS: AHI ≥ 30; BMI: body mass index; ESS: Epworth Sleep Score; SNOT-20: Sinonasal Outcome Test-20; SaO2: arterial oxygen saturation; MCA_2_: mean of the second minimal cross-sectional area; CT: computed tomography; ^a^Chi-Square test; ^b^Kruskal-Wallis test; ∗P < 0.05.

**Table 2:** Comparison of characteristics before and after FESS when classified by apnea-hypopnea index (AHI).

Classification	Non-OSAS			Mild OSAS			Moderate OSAS			Severe OSAS		
	N= 32			N= 26			N= 26			N= 12		

	Pre-FESS	Post-FESS	P ^a^	Pre-FESS	Post-FESS	P ^a^	Pre-FESS	Post-FESS	P ^a^	Pre-FESS	Post-FESS	P ^a^
SNOT-20 score	40.0±17.5	19.5±14.7	<0.001^∗^	37.9±18.7	20.4±16.0	<0.001^∗^	30.3±21.2	20.4±18.5	0.004^∗^	23.1±14.6	23.6±20.6	1.000
Nasal obstruction score	3.4±1.2	1.8±1.3	<0.001^∗^	3.4±1.6	1.2±1.1	<0.001^∗^	2.5±1.6	1.7±1.4	0.004^∗^	2.3±1.7	1.0±1.5	0.01^∗^
MCA_2_	0.36±0.17	0.46±0.23	0.004^∗^	0.40±0.15	0.49±0.19	0.033^∗^	0.42±0.20	0.49±0.21	0.048^∗^	0.49±0.19	0.55±0.15	0.387
Endoscopic score	5.1±1.9	4.9±1.9	0.615	5.2±2.3	4.4±2.0	0.036^∗^	5.5±2.4	4.3±1.7	0.001^∗^	5.1±3.0	4.9±2.0	0.887
BMI (kg/m^2^)	24.00±4.49	23.62±3.11	0.738	25.30±3.04	25.49±2.88	0.136	25.66±5.83	25.77±5.60	0.566	29.34±4.21	29.40±4.20	0.893
ESS score	8.9±5.2	6.5±4.2	0.007^∗^	8.4±4.6	6.6±4.1	0.034^∗^	9.2±4.5	7.2±4.0	0.012∗	5.7±3.7	6.7±5.8	0.349
Sleep domain of SNOT-20	14.2±7.8	7.0±6.7	<0.001^∗^	14.4±8.2	8.4±6.8	0.001^∗^	11.3±8.3	8.3±8.5	0.067	10.1±7.9	10.7±9.7	0.562
Snoring index	130.05±103.02	135.33±129.06	0.926	274.44±102.06	198.47±122.36	0.004^∗^	322.73±123.70	272.97±129.17	0.200	497.86±137.30	498.72±117.92	0.695
AHI	1.89±1.35	4.50±4.93	0.009^∗^	9.05±2.85	10.14±8.08	0.741	19.88±3.62	16.55±8.31	0.035^∗^	51.21±14.93	45.69±19.90	0.136
Lowest SaO_2_ (%)	90.53±3.06	90.16±3.14	0.595	86.19±5.10	86.15±5.21	0.903	85.14±7.73	84.92±6.64	0.444	72.68±12.53	72.50±12.46	0.624

FESS: functional endoscopic sinus surgery; OSAS: obstructive sleep apnea syndrome; Non-OSAS: AHI < 5; mild OSAS: AHI ≥ 5 but < 15; moderate OSAS: AHI ≥ 15 but < 30; severe OSAS: AHI ≥ 30; BMI: body mass index; ESS: Epworth Sleep Score; SNOT-20: Sinonasal Outcome Test-20; SaO_2_: arterial oxygen saturation; MCA_2_: mean of the second minimal cross-sectional area;  ^a^ Wilcoxon signed rank test; ∗P < 0.05.

**Table 3:** Preoperative characteristics of patients whose OSAS was successfully treated or not after FESS.

	Total (n=64)	Successful treatment of OSAS after FESS		P value
		AHI≥5 (n=55)	AHI<5 (n =9)	

Gender (Male/Female)	40/24	36/19	4/5	0.277 ^a^
Age (years)	47.97±13.74	48.91±13.93	42.22±11.56	0.127 ^b^
SNOT-20 score	32.00±19.61	31.64±19.73	34.22±19.91	0.685 ^b^
Preoperative nasal obstruction score	2.84±1.64	2.67±1.64	3.89±1.27	0.027∗ ^b^
MCA_2_	0.43±0.18	0.43±0.19	0.41±0.16	0.922 ^b^
Endoscopic score	5.33±2.44	5.25±2.37	5.78±2.95	0.624 ^b^
Without polyp/with polyp	34/30	31/24	3/6	0.285 ^a^
CT score	11.59±5.55	11.78±5.27	10.44±7.28	0.516 ^b^
BMI (kg/m^2^)	26.20±4.75	26.54±4.87	24.17±3.46	0.179 ^b^
ESS score	8.20±4.53	8.13±4.50	8.67±4.92	0.684 ^b^
Sleep-domain of SNOT-20	12.31±8.25	11.93±8.10	14.67±9.27	0.450 ^b^
Snoring index	335.95±141.86	352.46±141.05	235.04±104.22	0.016∗ ^b^
AHI score	21.35±16.75	23.36±17.21	9.11±3.62	0.001∗ ^b^
Lowest SaO2 (%)	83.23±9.37	82.53±9.82	87.56±3.94	0.146 ^b^

OSAS: obstructive sleep apnea syndrome; FESS: functional endoscopic sinus surgery; BMI: body mass index; ESS: Epworth Sleep Score; SNOT-20: Sinonasal Outcome Test-20; AHI: apnea-hypopnea index; SaO2: arterial oxygen saturation; MCA_2_: mean of the second minimal cross-sectional area; CT: computed tomography;  ^a^Fisher's Exact test;  ^b^Mann-Whitney U test; ∗P <0.05.

**Table 4:** Logistic regression analyses of predictors of successful treatment of OSAS after FESS (AHI <5).

Predictors	Univariate analysis			Multivariate analysis		
	OR	95 % CI	P value	OR	95 % CI	P value

Gender (Male/Female)	0.42	(0.101-1.760)	0.236	1.46	(0.235-9.023)	0.687
Age (years)	0.96	(0.913-1.017)	0.180	0.99	(0.925-1.050)	0.651
SNOT-20 score	1.01	(0.972-1.043)	0.712			
Nasal obstruction score	1.77	(0.997-3.140)	0.051	1.57	(0.831-2.980)	0.164
MCA_2_	0.50	(0.009-28.741)	0.735			
Endoscopic score	1.09	(0.820-1.451)	0.550			
Without polyp/with polyp	2.58	(0.585-11.403)	0.210			
CT score	0.96	(0.841-1.089)	0.503			
BMI (kg/m^2^)	0.84	(0.660-1.063)	0.144			
ESS score	1.03	(0.879-1.199)	0.739			
Sleep-domain of SNOT-20	1.04	(0.958-1.127)	0.357			
Snoring index	0.99	(0.986-0.999)	0.027∗			
AHI	0.79	(0.663-0.950)	0.012∗	0.78	(0.623-0.973)	0.028∗
Lowest SaO_2_ (%)	1.10	(0.968-1.241)	0.148			

OSAS: obstructive sleep apnea syndrome; FESS: functional endoscopic sinus surgery; BMI: body mass index; ESS: Epworth Sleep Score; SNOT-20: Sinonasal Outcome Test-20; AHI: apnea-hypopnea index; SaO2: arterial oxygen saturation; MCA_2_: mean of the second minimal cross-sectional area; CT: computed tomography; OR: Odds Ratio. ∗P<0.05.

**Table 5:** Liner regression analyses for association of changes in sleep outcomes, BMI, and rhinological parameters (after surgery-before surgery).

	ESS				Snoring index				AHI				Lowest SpO2			
	B	SE	Beta	P	B	SE	Beta	P	B	SE	Beta	P	B	SE	Beta	P

(Constant)	-0.47	0.59		0.428	-26.94	20.64		0.195	-0.98	1.14		0.390	-0.43	0.71		0.549
SNOT-20	0.09	0.03	0.36	0.001∗	0.42	0.91	0.05	0.648	-0.05	0.05	-0.11	0.304	0.01	0.03	0.04	0.741
BMI	-0.14	0.31	-0.05	0.653	6.94	10.71	0.07	0.519	0.03	0.59	0.01	0.961	-0.59	0.37	-0.17	0.114
MAC2	2.86	2.63	0.11	0.279	16.78	91.55	0.02	0.855	-5.93	5.04	-0.12	0.243	4.19	3.16	0.14	0.188

Adjusted R square	9.9%				-2.4%				-0.9%				1.3%			
F	4.35				0.27				0.72				1.39			
P value	0.007∗				0.844				0.540				0.251			

BMI: body mass index; ESS: Epworth Sleep Score; SNOT-20: Sinonasal Outcome Test-20; AHI: apnea-hypopnea index; SaO2: arterial oxygen saturation; MCA_2_: mean of the second minimal cross-sectional area; ∗*P *< 0.05.

## Data Availability

The data used to support the findings of this study are available from the first author upon request.
